# Representation of the Structure—A Key Point of Building QSAR/QSPR Models for Ionic Liquids

**DOI:** 10.3390/ma13112500

**Published:** 2020-05-30

**Authors:** Anna Rybińska-Fryca, Anita Sosnowska, Tomasz Puzyn

**Affiliations:** 1 Laboratory of Environmental Chemometrics, Faculty of Chemistry, University of Gdańsk, ul. Wita Stwosza 63, 80-308 Gdańsk, Poland; a.rybinska@qsarlab.com; 2 QSAR Lab Ltd., al. Grunwaldzka 190/102, 80-266 Gdańsk, Poland; a.sosnowska@qsarlab.com

**Keywords:** ionic liquids, QSAR, biological activity, molecular descriptors

## Abstract

The process of encoding the structure of chemicals by molecular descriptors is a crucial step in quantitative structure-activity/property relationships (QSAR/QSPR) modeling. Since ionic liquids (ILs) are disconnected structures, various ways of representing their structure are used in the QSAR studies: the models can be based on descriptors either derived for particular ions or for the whole ionic pair. We have examined the influence of the type of IL representation (separate ions vs. ionic pairs) on the model’s quality, the process of the automated descriptors selection and reliability of the applicability domain (AD) assessment. The result of the benchmark study showed that a less precise description of ionic liquid, based on the 2D descriptors calculated for ionic pairs, is sufficient to develop a reliable QSAR/QSPR model with the highest accuracy in terms of calibration as well as validation. Moreover, the process of a descriptors’ selection is more effective when the possible number of variables can be decreased at the beginning of model development. Additionally, 2D descriptors usually demand less effort in mechanistic interpretation and are more convenient for virtual screening studies.

## 1. Introduction

Ionic liquids (ILs) create a wide group of chemicals built of varied types of cations and anions. Their characteristic properties (e.g., melting point less than 100 °C; low vapor pressure; stability at wide range of temperatures; ability to serve as good solvents for various compounds) that can be precisely adjusted by structural modifications of particular ions make them a promising group of chemical materials [1]. They have found applications in different fields such as electrochemistry, separation and extraction techniques, synthesis, catalysis and biomass processing. However, since there is around a billion (10^12^) of potential binary (anions/cations) combinations, experimental optimization of ILs properties would be expensive and time consuming. Nevertheless, the selection of an ionic liquid having the optimal combination of the required properties is achievable by applying computational techniques such as the quantitative structure-activity/property relationship (QSAR/QSPR) approach [2]. QSAR/QSPR provides an opportunity to predict the property of interest for a number of empirically untested ILs based on the previously defined relationship between the variation in their chemical structures (encoded by a series of numerical values, so-called ‘descriptors’, e.g., the number of double bonds in the molecule) and the property (e.g., density, viscosity, octanol-water partition coefficient). The same applies for predicting biological activity, including toxicity (e.g., toxicity to *Vibrio fisheri*, *Daphnia magna* and *Danio rerio*), which is important from the human and environmental safety point of view [3,4,5,6]. By exploring the predictions coming from QSAR/QSPR models one is able to perform virtual screening of a vast number of ionic liquids to find ones with the preferred physicochemical properties and low toxicity to human and to the environment.

**Figure 1:**
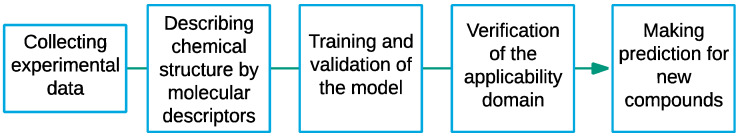
Basic steps that form the process of the quantitative structure-activity/property relationship (QSAR/QSPR) model development.

In general, the process of building a QSAR/QSPR model is based on five steps (Figure 1). However, in the case of ionic liquids, the way of representing the chemical structure by appropriately calculated molecular descriptors (the second step in Figure 1) is critical for the further model development [7]. Frequently, both ions (cation and anion) that consist of the IL are described separately. Moreover, the majority of the published models utilize the three-dimensional (3D) descriptors (descriptors that reflect 3D features of the molecule, e.g., solvent accessible surface area, molecular volume). For example, the QSPR model for predicting critical micellization concentration developed by Barycki et al. [8] is a linear combination of three descriptors: two of them (H8e and R7p+) characterize the structure of cation and one (HTi) describes the anion. In this case, the authors separately constructed and then optimized geometries of anions and cations structures to be used in the next step for calculating 3D descriptors. It is worth noting that the geometry optimization of molecular structures to be used for calculating descriptors is usually performed with quantum-chemical methods at a selected level of the theory. Barycki et al. [8] utilized the semi-empirical PM7 method. In our previous contribution [9], we investigated how the selection of the optimization method affects the 3D molecular descriptors (calculated separately for the anionic and cationic moieties) by considering three levels of the theory, namely: (i) semi-empirical with PM7 Hamiltonian (PM7), ab initio Hartree–Fock with 6-311 + G* basis set (HF/6-311 + G*) and density functional theory (DFT) with B3LYP hybrid functional and 6-311 + G* basis set (B3LYP/6-311 + G*). We proved that the descriptor values were dependent on the applied theory level. Moreover, we developed the respective QSPR models with use of the descriptors derived from the structures optimized at the three theory levels and then compared differences in the quality measures. We noticed that QSPR models utilizing descriptors calculated from the molecular geometries optimized at the level of PM7 and HF had similar values of the validation parameters (high values of the Q^2^ validation coefficient and low values of the root mean square error calculated for the external validation set), hence similarly good quality. In contrary, the model utilizing descriptors calculated from DFT-based geometries showed lower quality. The above results allowed the authors to recommend the use of the semi-empirical PM7 method as a routine for separate geometry optimization of anion and cation and then for the calculation of descriptors for anions and cations separately [9]. Subsequently, the two blocks of descriptors (calculated for different anions and cations) can be put together to form a single table of descriptors that characterizes the set of ILs (rows in the table correspond to particular ILs, whereas columns contain descriptors).

An alternative approach is to calculate molecular descriptors for the ionic pair. In that case, the structure of an ionic liquid is represented by molecular descriptors calculated as a sum of descriptors for the anion and the cation weighted by the molar fraction of each ion (the additive scheme) [10]. Geometries of both the anion and the cation are optimized separately. This scheme might be useful especially in the case of modeling ‘gemini’ ionic liquds. Moreover, the descriptors can be calculated not only from separately optimized ions, but also from the optimized ionic pair. Finally, it would be beneficial to replace 3D descriptors that require the molecular geometry to be optimized with much simpler and less time-consuming 2D descriptors that can be derived from two-dimensional representation of the structure (e.g., from a chemical structural formula). This, however, should be done without significant loss of QSAR/QSPR model’s quality.

Therefore, one of the crucial questions, when developing QSARs/QSPRs for ILs, is: How the chemical structure of an ionic liquid should be represented in order to obtain the most reliable QSAR/QSPR model? In this work we are trying to answer this question by performing a benchmark study to investigate advantages as well disadvantages of different approaches of describing the structure of ionic liquids. For the case study we have chosen a dataset that concerns toxicity towards *Esherichia coli*. However, we are convinced that similar conclusions could be drawn for the physicochemical dataset as well. We do believe that the presented results would serve as a starting point for further discussion on the development of QSAR/QSPR models for ionic liquids in order to accurately predict the physicochemical properties and biological activity of these compounds.

## 2. Materials and Methods 

### 2.1. Experimental Data and Molecular Descriptors

The experimental data of ILs’ toxicity towards *Escherichia coli* were collected from the literature [11]. The analyzed dataset contains 24 ionic liquids, in which six various types of cations (imidazolium, pyridinium, pyrrolidinium, piperidinium) and three anions (bromide, thiocyanate, bis(trifluoromethylsulfonyl)amide) can be distinguished. Antimicrobial activity was expressed as EC50 in mM unit. Detailed information can be found in the Table S1 in Supplementary Materials.

The structure of each ionic liquid has been described using molecular descriptors in two ways. The first set of descriptors contained those calculated after independent geometry optimization of each ion (A|B). The second one contained molecular descriptors calculated for the whole ionic liquid after geometry optimization of the ionic pair ([A+B]) by one of the DFT methods (B3LYP/6-311 + G*) with the Gaussian 09 software (Revision D.01, Gaussian, Inc., Wallingford, CT, USA) [12]. In the case of all sets, the descriptors values were calculated with the DRAGON (v. 7) software [13]. However, to increase efficiency of the benchmark study, the pool of descriptors was reduced to the following groups: constitutional descriptors, topological indices, ETA indices, walk and path counts, information indices, atom-centered fragments, WHIM, GETAWAY and Randic molecular profiles connected to molecular shape, and geometrical descriptors [14].

### 2.2. Model Development

The optimal, physically interpretable combination of the descriptors was selected by employing a stepwise selection algorithm in *olsrr* package implemented in R programming language [15]. The algorithm starts with an empty model. Then, in each step the best model (according to a specific criterion, e.g., lowest mean absolute error (MAE) value) is chosen from all models with one additional feature and from all models with one feature less. The algorithm was used for the sets of descriptors mentioned in previous section ((A|B) and [A + B]). The multiple linear regression (MLR) technique was used to find the relationship between the chemical structure of ionic liquids (described by molecular descriptors) and the modeled value (logEC50). Goodness-of-fit of the QSPR models was measured by using sets of measures such as the determination coefficient (R^2^), root mean square error of calibration (RMSE_C_), mean of absolute errors (MAE). All calculated metrics can be found in Supplementary Materials (Tables S2–S7).

### 2.3. Validation Process

According to requirements established by the Organization for Economic Co-operation and Development (OECD) referring to principles for the validation of QSAR models, we performed the internal and external validation of our models [16]. The stability of the models was verified by leave-one-out cross-validation coefficient (Q^2^_CV_) and root mean square error of cross-validation (RMSE_CV_). We also estimate the predictive ability by calculating two external validation measures: external validation coefficient (Q^2^_EXT_) and root mean square error of prediction (RMSE_EXT_). It should be noticed that external measures are calculated only for chemicals from the validation set. Additional parameters have been also calculated in order to confirm quality of the developed QSPR models, namely: concordance correlation coefficient (CCC) and modified r^2^ for whole dataset (r^2^_(overall)_) [17]. We also estimated the presence of influential points in the training set by performing F-test proposed by Toth et al., where F value is equal to: (1 – Q^2^_CV_)/(1 – R^2^) [18]. Moreover, we calculated other metrics and compared them with criteria proposed by Tropsha and thereby confirmed the good quality of the developed QSPR models [19]. Those criteria and values of all additional metrics can be found in Supplementary Materials (Tables S2–S7).

### 2.4. Applicability Domain

An essential part of the model development is related to the verification of the applicability domain (AD), which is defined as “the physico-chemical, structural, or biological space, knowledge or information on which the training set of the model has been developed, and for which it is applicable to make predictions for new compounds” [20]. Determining the applicability domain allows for estimating the reliability of predicted values (their interpolation or extrapolation), and thus verifies the model’s usefulness for new compounds. To define the applicability domain of our models we employed the standardization approach proposed by Roy et al [21]. As a result, we were able to identify compounds that could be considered as X-outliers or points outside of AD. Obtained results were compared with the leverage approach (Williams plot) [22]. In that approach the applicability domain is limited by the two critical values: three standard deviation units of the standardized residuals (±3σ) and the threshold leverage value (h*). The value of h* is calculated as h* = 3p’/n, where p’ is the number of model’s variables plus one, and n is the number of compounds in the training set. The predictions for compounds with h_i_ > h* are treated as the results of extrapolation, so they will be less reliable [23].

## 3. Results and Discussion

### 3.1. Relationship between the Form of Structure Representation and the Model Quality

In this part of the study we have investigated the influence of the structure representation on the model quality. The MLR method combined with the forward selection of descriptors was used to describe the relationship between the structure of ionic liquids and its toxicity towards *E. coli* bacterium. The six model equations (Table 1, M1–M6) were developed, to examine how the way of describing the ILs structure influences the quality of the QSAR model. The first three models (M1–M3) were built using 2D and 3D descriptors calculated for each of the cations and anions separately, after the geometry optimization of a particular ion. The remaining ones (M4–M6) were developed with 2D and 3D descriptors calculated from the optimized geometries of whole ionic pairs. 

The six models utilized two or three, uncorrelated descriptors: Psi_i_0—intrinsic state pseudoconnectivity index, type 0; SMTIV—Schultz MTI by valence vertex degrees; L1m—1st component size directional WHIM index/weighted by mass; L1i—1st component size directional WHIM index/weighted by ionization potential; QZZm—quadrupole z-component value/weighted by mass; GMTI—Gutman molecular topological index; MDDD—mean distance degree deviation; AMW—average molecular weight; L/Bw—length-to-breadth ratio by WHIM; RTv—R total index/weighted by van der Waals volume; L3u—3rd component size directional WHIM index/unweighted; E1e—1st component accessibility directional WHIM index/weighted by Sanderson electronegativity; DISPm—displacement value/weighted by mass. Superscripts A and C in equation stands for anion, cation respectively. The absence of a superscript means that the descriptor was calculated for the ionic pair.

**Figure 2:**
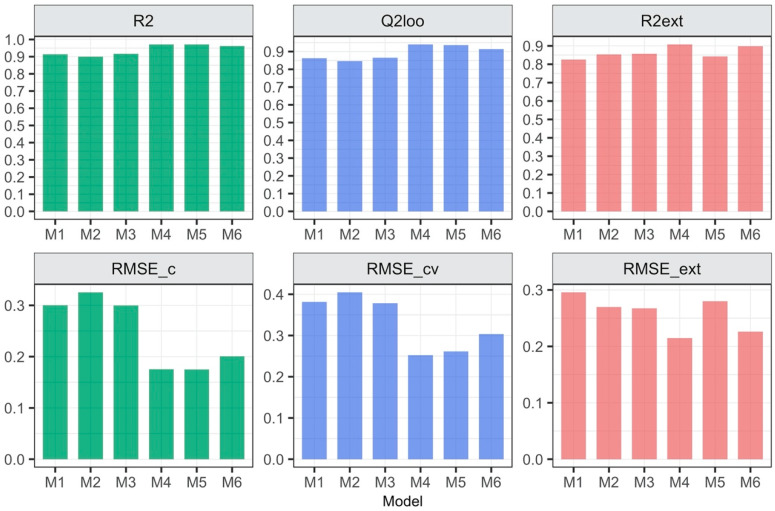
Quality measurements of the developed QSAR models.

**Figure 3:**
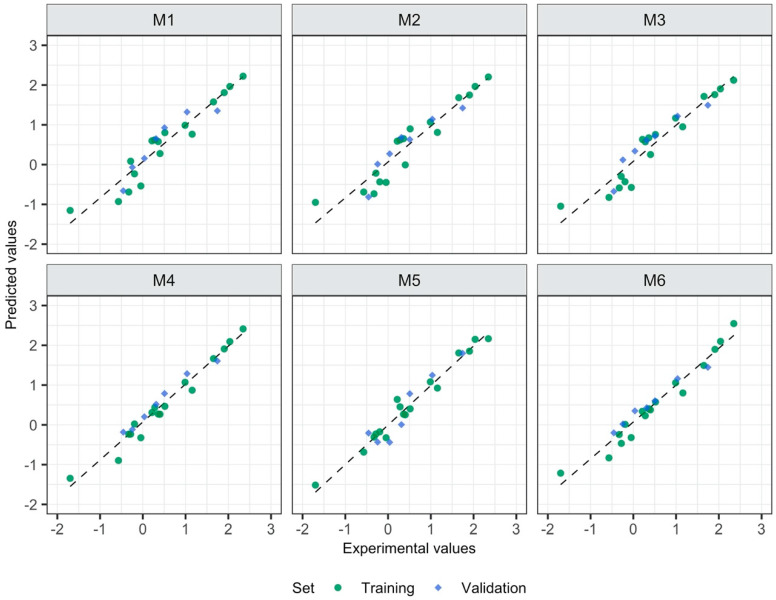
Experimental vs. predicted toxicity towards *E. coli* (log EC_50_, (mM)).

All models are characterized by satisfactory goodness-of-fit, robustness and predictive capabilities (the values of R^2^, Q^2^_CV_, Q^2^_EXT_ close to 1 and low values of the errors: RMSE_C_, RMSE_CV_, RMSE_EXT_) (Figure 1). Surprisingly, models with 3D descriptors are not the ones with the best quality metrics. The model based on 2D descriptors calculated for ionic pair (M4) is the one that is most accurate in terms of internal as well as external data set. The visual correlations between the experimental and the predicted log EC50 values for all developed models confirmed the differences in the statistical parameters mentioned above (Figure 2 and Figure 3).

When the tool for applicability domain (AD) evaluation recently developed by Roy et al. [21] was applied, none of the ILs were classified as an object out of the domain. The same conclusions can be derived from the leverage approach (a standard approach used for AD evaluation). For all models, the residual values for all training and validation ILs were within ±3 standard deviations from the mean value. Thus, the perditions were correct in relation to the molecular structures’ variation. However, there were several ILs with high leverage values, considered as “good leverage points”, that stabilized the models [21,22]. Details on the AD evaluation can be found in Supplementary Materials (Tables S2–S7).

Among the models based on descriptors calculated for separate ions (M1–M3), the one utilizing 2D and 3D descriptors (M3) can be considered as “the best” in terms of quality parameters. The first descriptor present in the equation (Psi_i_0^A^) reflects the electronegativity of atoms in the molecule and its topology (anions in this case) [24], whereas the second (QZZm^C^) characterizes the distribution of electric charges, taking into account the mass of the cation. 

In the case of the models utilizing descriptors calculated for ionic pairs (M4–M6), the one with 2D descriptors only (M4) is the most accurate. It employs three descriptors: GMTI that describes the structure branching; MDDD reflecting the molecular size; and AMW—the average molecular weight related to the atomic composition.

The most accurate models from both groups (namely: M3 and M4) have comparable values of the quality measures. However, QSAR models should not only be well described by statistical parameters, but also be interpretable in relation to the toxicity mechanism. In the case of M3, one can directly analyze, which ionic moiety (anion or cation) has a bigger impact on the toxicity towards *E. coli*. Unfortunately, the descriptors selected to the equation are not intuitive and easy for interpretation by non-experts. On the contrary, mechanistic interpretation of descriptors chosen for the M4 equation is simpler. Although all of the selected descriptors refer to the molecular (or ionic) size and shape, there are some important differences: descriptors in M3 account topology of the molecule as well as molecular properties such as electronegativity, whereas descriptors in M4 characterize the size and shape in a straighter way. Previous studies proved that factors such as branching (long alkyl chains) as well as molecular volume determine the lipophilic interactions. Thus, they can influence the toxicity of ILs towards *E. coli* [25,26,27]. Molecular descriptors in the M4 model equation are reflecting those factors. Therefore, we can conclude that the developed model (M4) is not a random correlation but is consistent with the existing knowledge of the studied toxicity mechanism.

The performed case study demonstrated that the way of molecular structure representation influences not only the quality of the model but also the possibility of interpretation. Both aspects should be considered during QSAR model development to obtain a reliable tool for hazard assessment of ionic liquids.

### 3.2. Influence of Structure Representation on the Variable Selection

In the second section, we studied the consequences of changing the representation approach on the descriptors’ selection. Different ways of calculating descriptors (see Section 3.1) resulted in different numbers of descriptors to be considered at the stage of building the predictive QSAR models (Table 2). For example, in the case of M1, every ionic liquid was initially described by 278 anionic and 294 cationic 2D descriptors. In the case of M4, the same ILs were also described by 2D descriptors, but since they encoded the structure of the ionic pair (as a sum of descriptors calculated for particular ions), their number (298) is almost two times lower. When 3D descriptors were added, the total number of descriptors to be considered when selecting variables to the model increased significantly. It is worth to mention that our case study was based on the limited types of descriptors. The maximum number of descriptors would even reach several thousand, if all types of descriptors available in the modern software (e.g., DRAGON, alvaDesc) were calculated. Preserving the highest possible ratio between the number of objects (here: ionic liquids) in the training set and variables (here: descriptors) is crucial for the efficient execution of the feature selection algorithms. Moreover, any feature selection algorithm (e.g., genetic algorithm) will be more effective and time-competitive when working on a smaller set of possibly important descriptors [28].

The study was performed in two groups of models, dependently on the way of description of the analyzed ionic liquids: (i) models in which ions are described separately (M1–M3) and (ii) models that utilized descriptors calculated for the whole ionic pair (M4–M6). For the purpose of the benchmark study, we have chosen stepwise selection method, a simple feature selection algorithm. In spite of using the identical control parameters, there are no common descriptors selected for models within both groups. The most probable explanation is the significant difference in the variance of variables. Moreover, models from the first group have similar values of the quality parameters to those from the second group despite the lower number of variables in the equation. Therefore, by using simpler way of ionic liquid representation, we were able to develop a model with high accuracy and a lower chance to be overfitted.

### 3.3. Influence of the Presence of the Second ion on Reliability of the Applicability Domain Assessment 

The main purpose of a QSAR model development is to create a tool that will deliver reliable predictions for new compounds. However, there is a critical condition: the new compound should belong to the applicability domain (AD) of the model. This means, its molecular structure should be similar to the training set enough to let the model interpolate (not extrapolate) the predicted endpoint value. Thus, majority of the AD assessment methods are based on the concept of molecular similarity. The algorithms of the molecular similarity calculation are based on the descriptors values used in the QSAR equation. This assumption is especially important in the case of models with 3D descriptors because their values could be influenced by the method selected to perform geometry optimization.

Obviously, when using descriptors calculated separately for the individual ions, the values of the descriptors are identical for all ionic liquids in the dataset. For example, thiocyanate anion has the same value of L1i^A^ descriptor (equals to 1.38) in the case of both: 1-butyl-3-methylpyridinium thiocyanate and 1-octyl-3-methylimidazolium thiocyanate. The same situation is in the case of cationic descriptors (Table 3). Therefore, the borders of the applicability domain are exact and easy to define. However, when the descriptors are calculated based on the structure of the whole ionic liquid the values of 3D descriptors are influenced by geometries of both ions. Thus, the same descriptors have a range of different values (Table 4). In consequence, the verification of AD is more challenging. Moreover, in such a case it should be investigated whether the selection of geometry optimization method and conditions would significantly affect the calculated 3D descriptors.

### 3.4. Other Practical Aspects of ILs Modeling with QSAR/QSPR

There are several examples in the literature, where QSAR models for ILs use even more sophisticated approaches of calculating the molecular descriptors than those presented in Section 3.1, Section 3.2 and Section 3.3. For example, Bruzzone et al. [29] developed a QSAR model for predicting toxicity (EC_50_) for *Vibrio fischeri* based on for 33 ionic liquids. Because all 33 ILs contained a halide anion (chloride or bromide), only the molecular structure of the cations was optimized at the DFT level of theory and used for calculating constitutional, topological, geometrical, electrostatic, and quantum chemical descriptors (with CODESSA software,). Similarly, Nekoeinia et al. [30] developed a QSPR model for predicting the normalized polarity parameter (E^T^_N_). The model was developed based on a set of 52 ILs having the same anions: (CF_3_SO_2_)N^2−^. The 2D and 3D descriptors such as topological and GETAWAY were calculated only for cations, after geometry optimization at the molecular mechanics level of theory (MM+ force field implemented in HyperChem software, v.7). In the model of cytotoxicity to the leukemia rat cell line (IPC-81) developed by Torrecilla et al. [31] the dataset included ionic liquids having various types of cation and anions. Therefore, the authors optimized molecular geometries of the cations and anions independently at the level of DFT (B3LYP/6-31++G**). Based on that, they derived the Sσ-profile molecular descriptors of counterions. A different approach was used by Wang et al. [32]. Although they modeled ionic liquids with the same anion (bromide), they were optimizing geometry of the whole structure (at the DFT level, B3LYP/6–311G (d, p)) in the case of each IL. Then, they used the quantum-mechanical properties (e.g., HOMO/LUMO energy, the total energy) of Br-ILs as descriptors in a QSAR model predicting toxicity towards *V. fischeri* and *D. magna*.

All the presented examples show that there are various ways of deriving structural information on IL encoded by molecular descriptors. Moreover, their values often depend on the method of geometry optimization. QSAR/QSPR models based on the 3D and/or quantum-chemical descriptors could provide deeper insight into the structure and properties of ionic liquids. However, a more precise description of the structure would not automatically guarantee better accuracy in predicting the endpoint value. Moreover, a wide use of QSAR methodology for finding new compounds with desired properties (e.g., viscosity, octanol-water partition coefficient, thermal stability) is possible on the condition that they are easy to apply and to reproduce by users not very experienced in computational chemistry. Thus, the necessity of performing geometry optimization of ions or ionic pairs with advanced computational techniques could limit the applicability of QSAR/QSPR modeling.

The reliable QSAR/QSPR model should have a well-defined endpoint and applicability domain, should be validated with an external data set, and should be assessed by correctly used statistical parameters [33]. However, QSAR models can be applied not only to predict the endpoint values for a large number of untested compounds, but also to investigate mechanisms of the observed toxicity. Thus, the descriptors employed in the model equation should enable to provide an interpretation of the possible mechanisms of toxicity [34]. Therefore, the selected combination of descriptors should be validated not only in terms of statistical requirements (i.e., goodness-of-fit), but also in the context of eventual sheading new light or consistency with the existing knowledge on the studied toxicity mechanism. Undoubtedly, the same aspect, i.e., the possibility to investigate mechanisms of the modeled property, has to be taken under consideration in terms of the development of QSPR models for predicting physicochemical properties.

## 4. Conclusions

This contribution was aimed at performing a benchmark study to investigate the relationship between the way of structure representation and the model quality. Moreover, we have discussed the advantages and disadvantages of several approaches of describing the structure of ionic liquids. The most important conclusions are:

- 2D descriptors are suitable to build reliable QSAR models;
- The strategy in which the 2D descriptors were calculated for the whole ionic liquid allowed to build the model with the highest quality;
- More precise description of the ionic liquid’s structure (through 3D descriptors calculated for ions or geometry optimization of ionic pairs followed by descriptors calculation) does not guarantee the better accuracy and predictive ability of the developed model;
- Models based on 2D descriptors are easier to apply and reproduce, even by non-experts in computational chemistry, which could lead to an increase of the application of in silico methods in various R&D areas.

Despite the fact that the case study concerns toxicity, we do believe that presented conclusions also concern the development of quantitative structure-property relationships (QSPR) models that allow predicting physicochemical properties.

## Figures and Tables

**Table 1:** The equations of all models developed in this study.

Model’s ID	Type of Descriptors	Descriptors Calculated for:	Equation for Predicting logEC_50_ [mM]
M1	2D	Separate ions	logEC_50_ = 2.49−0.14 Psi_i_0^A^ − 0.001 SMTIV^C^
M2	3D	Separate ions	logEC_50_ = 2.52−0.12 L1m^C^ − 0.19 L1i^A^
M3	2D, 3D	Separate ions	logEC_50_ = 2.304−0.142 Psi_i_0^A^ − 0.006 QZZm^C^
M4	2D	Ionic pair	logEC_50_ = 4.15−0.001 GMTI − 0.09 MDDD − 0.16 AMW
M5	3D	Ionic pair	logEC_50_ = 6.91−0.24 L/Bw − 1.05 RTv + 0.53 L3u
M6	2D, 3D	Ionic pair	logEC_50_ = 3.49−0.001 GMTI − 3.21 E1e + 0.04 DISPm

Superscripts A and C in equation stands for anion, cation respectively. The lack of superscript means that the descriptor was calculated for the ionic pair.

**Table 2:** Number of individual variables in investigated data sets.

Model	Number of All Variables	Anions’ Descriptors	Cations’ Descriptors
M1	572	278	294
M2	813	352	461
M3	1385	630	755
M4	298	0	0
M5	414	0	0
M6	712	0	0

**Table 3:** Three-dimensional (3D) descriptors calculated for anions and cations after separate optimization of ions. Descriptors in the table form equation of model M2.

IL	L1i^A^	L1m^C^	
[C4mpy] [SCN]	1.38	6.78	The same anion
[C8mim] [SCN]	1.38	18.4	
[C4mpyrr] [NTf2]	5.67	4.48	The same cation
[C4mpyrr] [Br]	0.00	4.48	

Superscripts A and C in equation stands for anion, cation respectively.

**Table 4:** Three-dimensional (3D) descriptors calculated for ionic pairs with the same anion present in M5 equation.

IL	L/Bw	RTv	L3u
[C4mim] [NTf2]	1.98	6.69	2.29
[C4py] [NTf2]	1.68	6.65	1.39
[C4mpyrr] [NTf2]	2.3	6.75	2.13
[C4mpip] [NTf2]	2.03	6.82	1.39
[C8mim] [NTf2]	2.85	7.20	2.13
[C8py] [NTf2]	2.2	7.16	1.56
[C8mpyrr] [NTf2]	2.05	6.93	1.28
[C8mpip] [NTf2]	2.23	7.34	1.90
Range	1.17	0.69	1.02

## Supplementary Materials

The following are available online at https://www.mdpi.com/1996-1944/13/11/2500/s1. Table S1: The experimental data of ILs’ toxicity towards *Escherichia coli* collected from the literature, Table S2: Details of the model based on 2D descriptors calculated for each of the cations and anions separately (M1), Table S3: Details of the model based on 3D descriptors calculated for each of the cations and anions separately (M2), Table S4: Details of the model based on 2D and 3D descriptors calculated for each of the cations and anions separately (M3), Table S5: Details of the model based on 2D descriptors calculated for whole ionic pairs (M4), Table S6: Details of the model based on 3D descriptors calculated from the optimized geometries of whole ionic pairs (M5), Table S7: Details of the model based on 2D and 3D descriptors from the optimized geometries of whole ionic pairs (M6).
