# Supplementary material for: Representation of the Structure—A Key Point of Building QSAR/QSPR Models for Ionic Liquids
Source: Materials (Basel). 2020 May 30;13(11):2500. doi: 10.3390/ma13112500 (PMC7321456; doi:10.3390/ma13112500)
Supplement: Supplementary file 1 [file materials-13-02500-s001.pdf]

materials

Article

Representation of the Structure—A Key Point of Building QSAR/QSPR Models for Ionic Liquids

Anna Rybińska–Fryca [1,2], Anita Sosnowska [2] and Tomasz Puzyn [1,2,*]

[1] Laboratory of Environmental Chemometrics, Faculty of Chemistry, University of Gdańsk, ul. Wita Stwosza 63, 80–308 Gdańsk, Poland; a.rybinska@qsarlab.com
[2] QSAR Lab Ltd., al. Grunwaldzka 190/102, 80–266 Gdańsk, Poland; a.sosnowska@qsarlab.com
* Correspondence: tomasz.puzyn@ug.edu.pl

Received: 30 April 2020; Accepted: 27 May 2020; Published: date

Table S1. The experimental data of ILs' toxicity towards Escherichia coli collected from the literature.

ID	Cation	Cation's Abbreviation	Cation's SMILES	Anion	Anion's Abbreviation	Anion's SMILES	Set	EC50 [mM]	log_EC50 [mM]
1	1−butyl−3−methylpyridinium	C4mpy	CCCC[N+]1=CC=CC(=C1)C	bromide	Br	[Br−]	T	45.12	1.65
2	1−butyl−3−methylpyridinium	C4mpy	CCCC[N+]1=CC=CC(=C1)C	thiocyanate	SCN	C(#N)[S−]	V	55.47	1.74
3	1−hexyl−3−methylpyridinium	C6mpy	CCCCCC[N+]1=CC=CC(=C1)C	bromide	Br	[Br−]	T	9.75	0.99
4	1−hexyl−3−methylpyridinium	C6mpy	CCCCCC[N+]1=CC=CC(=C1)C	thiocyanate	SCN	C(#N)[S−]	T	14.29	1.16
5	1−octyl−3−methylpyridinium	C8mpy	CCCCCCCC[N+]1=CC=CC(=C1)C	bromide	Br	[Br−]	V	1.09	0.04
6	1−octyl−3−methylpyridinium	C8mpy	CCCCCCCC[N+]1=CC=CC(=C1)C	thiocyanate	SCN	C(#N)[S−]	V	0.57	−0.24
7	1−decyl−3−methylpyridinium	C10mpy	CCCCCCCCCC[N+]1=CC=CC(=C1)C	bromide	Br	[Br−]	T	0.27	−0.57
8	1−decyl−3−methylpyridinium	C10mpy	CCCCCCCCCC[N+]1=CC=CC(=C1)C	thiocyanate	SCN	C(#N)[S−]	T	0.02	−1.70
9	1−ethyl−1−methylpyrrolidinium	C2mpyrr	CC[N+]1(CCCC1) C	bromide	Br	[Br−]	T	222.19	2.35
10	1−butyl−1−methylpyrrolidinium	C4mpyrr	CCCC[N+]1(CCCC1)C	bromide	Br	[Br−]	T	108.88	2.04
11	1−octyl−1−methylpyrrolidinium	C8mpyrr	CCCCCCCC[N+]1(CCCC1)C	bromide	Br	[Br−]	V	3.22	0.51
12	1−decyl−1−methylpyrrolidinium	C10mpyrr	CCCCCCCCCC[N+]1(CCCC1)C	bromide	Br	[Br−]	T	0.52	−0.28
13	1−butyl−1−methylpiperidinium	C4mpip	CCCC[N+]1(CCCCC1)C	bromide	Br	[Br−]	T	80.4	1.91
14	1−hexyl−1−methylpiperidinium	C6mpip	CCCCCC[N+]1(CCCCC1)C	bromide	Br	[Br−]	V	10.83	1.03
15	1−octyl−1−methylpiperidinium	C8mpip	CCCCCCCC[N+]1(CCCCC1)C	bromide	Br	[Br−]	T	1.91	0.28

16	1−octyl−3−methylimidazolium	C8mim	CCCCCCCCN1C=C[N+](=C1)C	thiocyanate	SCN	C(#N)[S−]	T	2.53	0.40
17	1−butyl−3−methylimidazolium	C4mim	CCCCN1C=C[N+](=C1)C	bis(trifluoromethylsulfonyl)amide	NTf2	C(F)(F)(F)S(=O)(=O)[N−]S(=O)(=O)C(F)(F)F	T	1.64	0.21
18	1−butylpyridinium	C4py	CCCC[N+]1=CC=CC=C1	bis(trifluoromethylsulfonyl)amide	NTf2	C(F)(F)(F)S(=O)(=O)[N−]S(=O)(=O)C(F)(F)F	T	2.3	0.36
19	1−butyl−1−methylpyrrolidinium	C4mpyrr	CCCC[N+]1(CCCC1)C	bis(trifluoromethylsulfonyl)amide	NTf2	C(F)(F)(F)S(=O)(=O)[N−]S(=O)(=O)C(F)(F)F	T	3.29	0.52
20	1−butyl−1−methylpiperidinium	C4mpip	CCCC[N+]1(CCCCC1)C	bis(trifluoromethylsulfonyl)amide	NTf2	C(F)(F)(F)S(=O)(=O)[N−]S(=O)(=O)C(F)(F)F	V	2.05	0.31
21	1−octyl−3−methylimidazolium	C8mim	CCCCCCCCN1C=C[N+](=C1)C	bis(trifluoromethylsulfonyl)amide	NTf2	C(F)(F)(F)S(=O)(=O)[N−]S(=O)(=O)C(F)(F)F	V	0.35	−0.46
22	1−octylpyridinium	C8py	CCCCCCCC[N+]1=CC=CC=C1	bis(trifluoromethylsulfonyl)amide	NTf2	C(F)(F)(F)S(=O)(=O)[N−]S(=O)(=O)C(F)(F)F	T	0.47	−0.33
23	1−octyl−1−methylpyrrolidinium	C8mpyrr	CCCCCCCC[N+]1(CCCC1)C	bis(trifluoromethylsulfonyl)amide	NTf2	C(F)(F)(F)S(=O)(=O)[N−]S(=O)(=O)C(F)(F)F	T	0.64	−0.19
24	1−octyl−1−methylpiperidinium	C8mpip	CCCCCCCC[N+]1(CCCCC1)C	bis(trifluoromethylsulfonyl)amide	NTf2	C(F)(F)(F)S(=O)(=O)[N−]S(=O)(=O)C(F)(F)F	T	0.9	−0.05

T—training set, V—validation set

Table S2. Details of the model based on 2D descriptors calculated for each of the cations and anions separately (M1).

Variable	Coeff.	Std. Error
Intercept	2.49	0.18
Psi_i_0[A]	−0.14	0.02
SMTIV[C]	−0.001	0.0001

Quality Parameters:

Fitting criteria	*Internal validation criteria*	*External validation criteria*

R^2	0.91	Q^2loo	0.86	R^2ext/Q^2–F1	0.83
$RMSE_C$	0.30	$RMSE_{CV}$	0.38	$RMSE_{EXT}$	0.30
R^2adj	0.90	R^2–Q^2loo	0.05	Q^2–F2	0.83
R^2–R^2adj	0.01	MAE_{CV}	0.31	MAE_{EXT}	0.28
MAE_C	0.25			r^2m aver.	0.76
RSS_C	1.53			r^2m delta	0.11
				r^2	0.85
				$r0^2$	0.84
				reverse $r0^2$	0.82

**Golbraikh and Tropsha Acceptable Model Criteria's:**			
1. Q^2	0.86	$Q^2 > 0.5$	Passed
2. r^2	0.85	$r^2 > 0.6$	Passed
3. $\mid r0^2 – r'^2 \mid$	0.03	$\mid r0^2 – r'0^2 \mid < 0.3$	Passed
4. k	0.89	[0.85 < k < 1.15 and ((r^2–r0^2)/r^2 < 0.1]	Passed
$[(r^2–r0^2)/r^2]$	0.01		
OR*			
k'	1.00	[0.85 < k' < 1.15 and ((r^2–$r'0^2$)/ r^2) < 0.1])	Passed
$[(r^2–r'0^2)/r^2]$	0.04		

**ID**	**Set**	**Psi_i_0**[A]	**SMTIV**[C]	**Exp. Endpoint**	**Pred. by Model eq.**	**Pred.Mod.Eq.Res.**	**Outlier or out of AD? (StdAD)**
1	T	0	900	1.65	1.58	0.08	No
3	T	0	1480	0.99	0.99	0.00	No
4	T	1.56	1480	1.16	0.76	0.39	No
7	T	0	3370	−0.57	−0.93	0.36	No
8	T	1.56	3370	−1.70	−1.15	−0.55	No
9	T	0	262	2.35	2.22	0.12	No
10	T	0	518	2.04	1.96	0.07	No

12	T	0	2370	−0.28	0.08	−0.37	No
13	T	0	669	1.91	1.81	0.10	No
15	T	0	1840	0.28	0.62	−0.34	No
16	T	1.56	1960	0.40	0.28	0.13	No
17	T	8.09	721	0.22	0.60	−0.38	No
18	T	8.09	740	0.36	0.58	−0.22	No
19	T	8.09	518	0.52	0.80	−0.29	No
22	T	8.09	1990	−0.33	−0.69	0.36	No
23	T	8.09	1540	−0.19	−0.23	0.04	No
24	T	8.09	1840	−0.05	−0.54	0.49	No
2	V	1.56	900	1.74	1.35	0.39	No
5	V	0	2300	0.04	0.16	−0.12	No
6	V	1.56	2300	−0.24	−0.07	−0.18	No
11	V	0	1540	0.51	0.93	−0.42	No
14	V	0	1150	1.04	1.32	−0.29	No
20	V	8.09	669	0.31	0.65	−0.34	No
21	V	8.09	1960	−0.46	−0.66	0.20	No

Table S3. Details of the model based on 3D descriptors calculated for each of the cations and anions separately (M2).

**Variable**	**Coef.**	**Std. Error**			
Intercept	2.52	0.20			
$L1m^C$	−0.12	0.01			
$L1i^A$	−0.19	0.03			
**Quality Parameters:**					
Fitting criteria		*Internal validation criteria*		*External validation criteria*	
R^2	0.90	Q^2loo	0.85	R^2ext/Q^2–F1	0.85

$RMSE_{C}$	0.33	$RMSE_{CV}$	0.40	$RMSE_{EXT}$	0.27
R^2adj	0.89	$R^2–Q^2loo$	0.05	$Q^2–F2$	0.85
$R^2–R^2adj$	0.01	MAE_{CV}	0.33	MAE_{EXT}	0.25
MAE_{C}	0.27			r^2m aver.	0.80
RSS_{C}	1.80			r^2m delta	0.10
				r^2	0.86
				$r0^2$	0.86
				reverse $r0^2$	0.85

Golbraikh and Tropsha Acceptable Model Criteria's:

1. Q^2	0.85	$Q^2 > 0.5$	Passed
2. r^2	0.86	$r^2 > 0.6$	Passed
3. $\lvert r0^2–r'0^2 \rvert$	0.01	$\lvert r0^2–r'0^2 \rvert < 0.3$	Passed
4. k	0.93		
[(r^2–r0^2)/r^2]	0.002	$[0.85 < k < 1.15$ and $((r^2–r0^2)/r^2 < 0.1]$	Passed
OR*			
k'	0.96		
$[(r^2–r'0^2)/r^2]$	0.02	$[0.85 < k' < 1.15$ and $((r^2–r'0^2)$/r^2) < 0.1])	Passed

**ID**	**Set**	**L1m[C]**	**L1i[A]**	**Exp. Endpoint**	**Pred. by Model eq.**	**Pred.Mod.Eq.Res.**	**Outlier or out of AD? (StdAD)**
1	T	6.779	0	1.65	1.68	−0.03	No
3	T	11.763	0	0.99	1.07	−0.08	No

4	T	11.763	1.378	1.16	0.81	0.35	No
7	T	26.051	0	−0.57	−0.69	0.12	No
8	T	26.051	1.378	−1.70	−0.95	−0.75	No
9	T	2.543	0	2.35	2.20	0.15	No
10	T	4.483	0	2.04	1.96	0.08	No
12	T	22.202	0	−0.28	−0.22	−0.06	No
13	T	6.231	0	1.91	1.75	0.16	No
15	T	15.419	0	0.28	0.62	−0.34	No
16	T	18.4	1.378	0.40	−0.01	0.41	No
17	T	6.996	5.665	0.22	0.59	−0.38	No
18	T	6.482	5.665	0.36	0.65	−0.29	No
19	T	4.483	5.665	0.52	0.90	−0.38	No
22	T	17.744	5.665	−0.33	−0.73	0.40	No
23	T	15.31	5.665	−0.19	−0.43	0.24	No
24	T	15.419	5.665	−0.05	−0.45	0.40	No
2	V	6.779	1.378	1.74	1.42	0.32	No
5	V	18.237	0	0.04	0.27	−0.23	No
6	V	18.237	1.378	−0.24	0.01	−0.26	No
11	V	15.31	0	0.51	0.63	−0.12	No
14	V	11.166	0	1.04	1.14	−0.11	No
20	V	6.231	5.665	0.31	0.68	−0.37	No
21	V	18.4	5.665	−0.46	−0.81	0.36	No

Table 4. Details of the model based on 2D and 3D descriptors calculated for each of the cations and anions separately (M3).

Variable	Coeff.	Std. Error			
Intercept	2.304	0.1653			
$Psi\_i\_0^{A}$	−0.142	0.0217			
$QZZm^{C}$	−0.006	0.0005			
**Quality Parameters:**					
Fitting criteria		*Internal validation criteria*		*External validation criteria*	
R^2	0.92	Q^2loo	0.87	$R^2ext/Q^2–F1$	0.86
$RMSE_C$	0.30	$RMSE_{CV}$	0.38	$RMSE_{EXT}$	0.27
R^2adj	0.905	$R^2–Q^2loo$	0.05	$Q^2–F2$	0.86
$R^2–R^2adj$	0.012	MAE_{CV}	0.32	MAE_{EXT}	0.26
MAE_C	0.25			r^2m aver.	0.75
RSS_C	1.50			r^2m delta	0.10
				r^2	0.89
				$r0^2$	0.87
				reverse r0^2	0.84
**Golbraikh and Tropsha acceptable model criteria's:**					
1. Q^2	0.86	$Q^2 > 0.5$	Passed		
2. r^2	0.89	$r^2 > 0.6$	Passed		
3. $\lvert r0^2–r'0^2 \rvert$	0.03	$\lvert r0^2–r'0^2 \rvert < 0.3$	Passed		
4. k	0.91	$[0.85 < k < 1.15$ and $((r^2–r0^2)/r^2 < 0.1]$	Passed		
$[(r^2–r0^2)/r^2]$	0.02				
OR*					
k'	0.99	$[0.85 < k' < 1.15$ and $((r^2–r'0^2)/r^2) < 0.1])$	Passed		

$[(r^2-r'0^2)/r^2]$	0.06

ID	Set	Psi_i_0^A	QZZm^C	Exp. Endpoint	Pred. by Model eq.	Pred.Mod.Eq.Res.	Outlier or out of AD? (StdAD)
1	T	0	99	1.65	1.71	−0.06	No
3	T	0	190.258	0.99	1.17	−0.18	No
4	T	1.56	190.258	1.16	0.95	0.21	No
7	T	0	525.383	−0.57	−0.83	0.26	No
8	T	1.56	525.383	−1.70	−1.05	−0.65	No
9	T	0	30.861	2.35	2.12	0.23	No
10	T	0	67.211	2.04	1.90	0.13	No
12	T	0	436.357	−0.28	−0.29	0.01	No
13	T	0	91.447	1.91	1.76	0.15	No
15	T	0	290.62	0.28	0.57	−0.29	No
16	T	1.56	307.266	0.40	0.25	0.15	No
17	T	8.09	87.871	0.22	0.63	−0.42	No
18	T	8.09	81.203	0.36	0.67	−0.31	No
19	T	8.09	67.211	0.52	0.76	−0.24	No
22	T	8.09	292.369	−0.33	−0.59	0.26	No
23	T	8.09	266.49	−0.19	−0.43	0.24	No
24	T	8.09	290.62	−0.05	−0.57	0.53	No
2	V	1.56	99	1.74	1.49	0.25	No
5	V	0	329.776	0.04	0.34	−0.30	No
6	V	1.56	329.776	−0.24	0.12	−0.36	No
11	V	0	266.49	0.51	0.72	−0.21	No
14	V	0	182.483	1.04	1.22	−0.18	No
20	V	8.09	91.447	0.31	0.61	−0.30	No

21	V	8.09	307.266	−0.46	−0.67	0.22	No

Table S5. Details of the model based on 2D descriptors calculated for whole ionic pairs (M4).

**Variable**	**Coeff.**	**Std. Error**			
Intercept	4.15	0.40			
GMTI	−0.001	0.0001			
MDDD	−0.09	0.02			
AMW	−0.16	0.04			
**Quality Parameters:**					
Fitting criteria		*Internal validation criteria*		*External validation criteria*	
R^2	0.97	Q^2loo	0.94	R^2ext/Q^2–F1	0.91
$RMSE_C$	0.18	$RMSE_{CV}$	0.25	$RMSE_{EXT}$	0.21
R^2adj	0.96	R^2–Q^2loo	0.03	Q^2–F2	0.91
R^2–R^2adj	0.01	MAE_{CV}	0.19	MAE_{EXT}	0.21
MAE_C	0.14			r^2m aver.	0.74
RSS_C	0.52			r^2m delta	0.07
				r^2	0.97
				$r0^2$	0.92
				reverse $r0^2$	0.89
**Golbraikh and Tropsha acceptable model criteria's:**					

1. Q^2	0.94	$Q^2 > 0.5$	Passed
2. r^2	0.97	$r^2 > 0.6$	Passed
3. $\lvert r0^2 – r'0^2 \rvert$	0.03	$\lvert r0^2 – r'0^2 \rvert < 0.3$	Passed
4. k	0.93	[0.85 < k < 1.15 and $((r^2 – r0^2)/r^2 < 0.1]$	Passed
$[(r^2 – r0^2)/r^2]$	0.06		
OR*			
k'	1.01	[0.85 < k' < 1.15 and $((r^2 – r'0^2)/r^2) < 0.1]$	Passed
$[(r^2 – r'0^2)/r^2]$	0.09		

ID	Set	GMTI	MDDD	AMW	Exp. Endpoint	Pred. by Model eq.	Pred.Mod.Eq.Res.	Outlier or out of AD? (StdAD)
1	T	609	7.17	8.22	1.65	1.66	−0.01	No
3	T	1080	10.3	7.59	0.99	1.07	−0.08	No
4	T	1090	14.1	6.57	1.16	0.87	0.29	No
7	T	2690	17.9	6.83	−0.57	−0.90	0.33	No
8	T	2690	24	6.09	−1.70	−1.34	−0.36	No
9	T	201	3.65	7.77	2.35	2.41	−0.06	No
10	T	435	5.95	7.17	2.04	2.09	−0.05	No
12	T	2230	16	6.25	−0.28	−0.23	−0.05	No
13	T	577	7	6.95	1.91	1.91	0.00	No
15	T	1700	13.5	6.36	0.28	0.43	−0.15	No
16	T	1490	17.3	6.34	0.40	0.27	0.13	No
17	T	1420	10.4	10.5	0.22	0.31	−0.10	No
18	T	1440	10.3	10.7	0.36	0.27	0.09	No
19	T	1390	10.9	9.39	0.52	0.46	0.06	No
22	T	2460	8.73	9.27	−0.33	−0.23	−0.10	No

23	T	2370	8.28	8.4	−0.19	0.02	−0.21	No
24	T	2660	9.65	8.21	−0.05	−0.32	0.27	No
2	V	615	9.92	6.95	1.74	1.60	0.14	No
5	V	1770	14	7.16	0.04	0.20	−0.17	No
6	V	1770	18.9	6.3	−0.24	−0.12	−0.13	No
11	V	1420	12.1	6.47	0.51	0.79	−0.28	No
14	V	1040	10	6.61	1.04	1.29	−0.25	No
20	V	1530	9.54	9.09	0.31	0.52	−0.21	No
21	V	2430	8.66	9.15	−0.46	−0.18	−0.27	No

Table S6. Details of the model based on 3D descriptors calculated from the optimized geometries of whole ionic pairs (M5).

Variable	Coeff.	Std. Error
Intercept	6.91	0.34
L/Bw	−0.24	0.02
RTv	−1.05	0.07
L3u	0.53	0.18

Quality Parameters:

Fitting criteria		*Internal validation criteria*		*External validation criteria*	
R^2	0.97	Q^2loo	0.94	R^2ext/Q^2–F1	0.84
$RMSE_{C}$	0.18	$RMSE_{CV}$	0.26	$RMSE_{EXT}$	0.28
R^2adj	0.96	R^2–Q^2loo	0.04	Q^2–F2	0.85
R^2–R^2adj	0.01	MAE_{CV}	0.20	MAE_{EXT}	0.25
MAE_{C}	0.14			r^2m aver.	0.77
RSS_{C}	0.52			r^2m delta	0.09
				r^2	0.89
				$r0^2$	0.88
				reverse $r0^2$	0.89

Golbraikh and Tropsha Acceptable Model Criteria's:

1. Q^2	0.94	$Q^2 > 0.5$	Passed
2. r^2	0.89	$r^2 > 0.6$	Passed
3. $\lvert r0^2 - r'0^2 \rvert$	0.01	$\lvert r0^2 - r'0^2 \rvert < 0.3$	Passed
4. k	0.86	$[0.85 < k < 1.15$ and $((r^2 - r0^2)/r^2 < 0.1]$	Passed
$[(r^2 - r0^2)/r^2]$	0.02		
OR*			Passed
k'	1.06	$[0.85 < k' < 1.15$ and $((r^2 - r'0^2)/r^2) < 0.1]$	Passed
$[(r^2 - r'0^2)/r^2]$	0.01		

ID	Set	L/Bw	RTv	L3u	Exp. Endpoint	Pred. by model eq.	Pred.Mod.Eq .Res.	Outlier or out of AD? (StdAD)
1	T	1.6	4.874	0.746	1.65	1.81	−0.16	No
3	T	4.37	5.002	0.903	0.99	1.08	−0.09	No
4	T	4.18	5.474	1.454	1.16	0.92	0.24	No
7	T	9.44	5.509	0.905	−0.57	−0.69	0.12	No
8	T	10.52	6.068	0.941	−1.70	−1.52	−0.18	No
9	T	3.76	4.232	1.147	2.35	2.17	0.18	No
10	T	1.59	4.725	1.094	2.04	2.15	−0.11	No
12	T	7.66	5.532	0.992	−0.28	−0.23	−0.05	No
13	T	1.29	5.175	1.284	1.91	1.85	0.06	No
15	T	4.61	5.671	1.151	0.28	0.45	−0.17	No
16	T	6.58	5.298	0.941	0.40	0.25	0.15	No
17	T	1.96	6.685	2.292	0.22	0.64	−0.43	No
18	T	1.68	6.651	1.392	0.36	0.27	0.09	No
19	T	2.3	6.749	2.127	0.52	0.40	0.12	No
22	T	2.2	7.163	1.557	−0.33	−0.31	−0.02	No
23	T	2.05	6.929	1.277	−0.19	−0.18	−0.01	No
24	T	2.23	7.341	1.896	−0.05	−0.32	0.27	No
2	V	2.8	4.732	1.003	1.74	1.80	−0.06	No
5	V	8.96	5.498	1.127	0.04	−0.44	0.48	No
6	V	7.86	5.778	1.185	−0.24	−0.43	0.19	No
11	V	4.56	5.3	1.018	0.51	0.78	−0.27	No
14	V	2.41	5.458	1.22	1.04	1.25	−0.21	No
20	V	2.03	6.818	1.391	0.31	0.01	0.31	No

21	V	2.85	7.201	2.126	−0.46	−0.21	−0.25	No

Table S7. Details of the model based on 2D and 3D descriptors from the optimized geometries of whole ionic pairs (M6).

**Variable**	**Coeff.**	**Std. Error**
Intercept	3.49	0.53
GMTI	−0.001	0.00
E1e	−3.21	0.81
DISPm	0.04	0.02

Quality Parameters:

Fitting criteria		*Internal validation criteria*		*External validation criteria*	
R^2	0.96	Q^2loo	0.91	R^2ext/Q^2–F1	0.90
$RMSE_C$	0.20	$RMSE_{CV}$	0.30	$RMSE_{EXT}$	0.23
R^2adj	0.95	R^2–Q^2loo	0.05	Q^2–F2	0.90
R^2–R^2adj	0.01	MAE_{CV}	0.22	MAE_{EXT}	0.21
MAE_C	0.16			r^2m aver.	0.65
RSS_C	0.68			r^2m delta	0.11
				r^2	0.98
				$r0^2$	0.90
				reverse $r0^2$	0.85

Golbraikh and Tropsha Acceptable Model Criteria's:

1. Q^2	0.91	$Q^2 > 0.5$	Passed
2. r^2	0.98	$r^2 > 0.6$	Passed
3. $\lvert r0^2 – r'0^2 \rvert$	0.05	$\lvert r0^2 – r'0^2 \rvert < 0.3$	Passed
4. k<br>$[(r^2–r0^2)/r^2]$	1.03<br>0.08	$[0.85 < k < 1.15$ and $((r^2–r0^2)/r^2 < 0.1]$	Passed
OR*			Passed

k'	0.90	[0.85 < k' < 1.15 and $((r^2 - r'^2_0)/r^2) <$ 0.1]	Passed
$[(r^2 - r'^2_0)/r^2]$	0.13		

ID	Set	GMTI	E1e	DISPm	Exp. Endpoint	Pred. by Model eq.	Pred.Mod.Eq.Res.	Outlier or out of AD? (StdAD)
1	T	609	0.528	11.096	1.65	1.49	0.16	No
3	T	1080	0.562	17.687	0.99	1.06	−0.07	No
4	T	1090	0.546	11.082	1.16	0.80	0.36	No
7	T	2690	0.568	23.257	−0.57	−0.83	0.26	No
8	T	2690	0.547	13.135	−1.70	−1.21	−0.49	No
9	T	201	0.446	16.767	2.35	2.55	−0.20	No
10	T	435	0.464	14.929	2.04	2.10	−0.06	No
12	T	2230	0.557	17.057	−0.28	−0.47	0.19	No
13	T	577	0.452	13.776	1.91	1.90	0.01	No
15	T	1700	0.52	14.36	0.28	0.23	0.05	No
16	T	1490	0.537	12.694	0.40	0.38	0.02	No
17	T	1420	0.622	15.915	0.21	0.34	−0.13	No
18	T	1440	0.616	17.52	0.36	0.40	−0.04	No
19	T	1390	0.616	19.885	0.52	0.57	−0.05	No
22	T	2460	0.454	21.417	−0.33	−0.24	−0.09	No
23	T	2370	0.41	21.32	−0.19	0.01	−0.20	No
24	T	2660	0.385	20.641	−0.05	−0.32	0.27	No
2	V	615	0.545	11.476	1.74	1.45	0.30	No
5	V	1770	0.579	23.399	0.04	0.35	−0.31	No
6	V	1770	0.553	14.124	−0.24	0.02	−0.26	No
11	V	1420	0.536	15.651	0.51	0.60	−0.10	No

14	V	1040	0.488	13.477	1.04	1.16	−0.13	No
20	V	1530	0.613	20.523	0.31	0.43	−0.12	No
21	V	2430	0.446	20.942	−0.46	−0.20	−0.26	No

© 2020 by the authors. Submitted for possible open access publication under the terms and conditions of the Creative Commons Attribution (CC BY) license (http://creativecommons.org/licenses/by/4.0/).
